# Geno- and seroprevalence of *Felis domesticus* Papillomavirus type 2 (FdPV2) in dermatologically healthy cats

**DOI:** 10.1186/s12917-016-0776-7

**Published:** 2016-07-22

**Authors:** Marco Geisseler, Christian E. Lange, Claude Favrot, Nina Fischer, Mathias Ackermann, Kurt Tobler

**Affiliations:** Institute of Virology. Vetsuisse Faculty, University of Zurich Winterthurerstrasse 266a, 8057 Zurich, Switzerland; Dermatology Department, Clinic for Small Animal Internal Medicine, Vetsuisse Faculty, University of Zurich, Winterthurerstrasse 260, 8057 Zurich, Switzerland; Present address: Amt für Landwirtschaft und Natur des Kantons Bern, Veterinärdienst, Herrengasse 1, 3011 Berne, Switzerland; Present address: Department of Microbiology and Immunobiology, Harvard Medical School, 77 Avenue Louis Pasteur, Boston, MA 02115 USA

**Keywords:** Cat, FdPV2, BISC, Papillomavirus, Prevalence

## Abstract

**Background:**

Papillomaviruses can cause proliferative skin lesions ranging from benign hyperplasia to squamous cell carcinoma (SCC). However, asymptomatic infection is also possible. Several groups have detected *Felis domesticus* Papillomavirus type 2 (FdPV2) DNA in association with feline Bowenoid in situ carcinoma (BISC). Therefore, a causative connection has been suggested. However, the knowledge about FdPV2 epidemiology is limited. The aim of this study was to describe the genoprevalence and seroprevalence of FdPV2 in healthy cats.

For this purpose an FdPV2-specific quantitative (q)PCR assay was developed and used to analyse Cytobrush samples collected from 100 dermatologically healthy cats. Moreover, an ELISA was established to test the sera obtained from the same cats for antibodies against the major capsid protein (L1) of FdPV2.

**Results:**

The genoprevalence of FdPV2 was to 98 %. Surprisingly, the quantities of viral DNA detected in some samples from the healthy cats exceeded the amounts detected in control samples from feline BISC lesions. The seroprevalence was much lower, amounting to 22 %. The concentrations of antibodies against FdPV2 were relatively low in healthy cats, whereas they were very high in control cats with BISC.

**Conclusion:**

These observations suggest that FdPV2 is highly prevalent, even among healthy cats. However, cats that carry it on their skin mount in most instances no antibody response. It might be hypothesized that FdPV2 is only rarely productively replicating or its replication is only rarely exposed to the immune system.

**Electronic supplementary material:**

The online version of this article (doi:10.1186/s12917-016-0776-7) contains supplementary material, which is available to authorized users.

## Background

Papillomaviruses (PVs) are small non-enveloped DNA viruses. They possess a double-stranded, circular genome of approximately 8 kilobasepairs (kbp), typically divided into an early (E) and late (L) region. The early regions encode viral regulatory proteins (E1, E2, E6, and E7) whereas the late regions encode the capsid proteins (L1 and L2). The capsid is of icosahedral shape and consists of major capsid protein L1, organised in 72 pentameric subunits, and minor capsid protein L2 [1].

PVs can be found in various higher vertebrates including mammals, birds and reptiles [2, 3]. Most host species can be infected by multiple different PV species and types but for few exceptions, PVs tend to be highly species specific [1, 4–6]. By 2007, seven PVs specific for *Felidae* were described. They were found in six different animal species, namely *Felis catus*, *Puma concolor* (Cougar), *Lynx rufus* (Bobcat), *Panthera leo* (Lion), *Neofelis nebulosa* (Clouded Leopard), and *Unica unica* (Snow Leopard) [7, 8]. All *Felidae* PVs that were actually sequenced at that time were classified into the genus *Lambdapapillomavirus* [2] and results from phylogenetic analysis proposed a coevolution of these viruses with their hosts [9].

The first partial sequences of the second feline PV were reported in 2006 [10]. After sequencing its whole genome in 2007, it was named feline PV type 2 (FdPV2) and classified into the newly created genus *dyo-Thetapapillomavirus* [2, 11]*.* The virus is also referred to as Felis catus PV type 2 (FcaPV2). Meanwhile, the number of PVs specific for the domestic cat has increased to four [2, 12, 13]. Recently, BPV14 isolated from a domestic cat suffering from feline sarcoid was sequenced on its entire genome length [14]. A cross-species infection of BPV14 in cats was therefore suggested.

Clinically, PVs show a specific cellular tropism for squamous epithelial cells [1]. They can cause proliferative lesions ranging from benign warts to squamous cell carcinoma (SCC) [1, 2]. Initially, FdPV2 DNA had been solely detected in feline Bowenoid in situ carcinomas (BISC). BISC is a rare premalignant state of SCC [15, 16]. BISC is a non-painful, pigmented, plaque like lesion within the haired skin. It can occur at any site of the body and there are usually multiple ones. In some BISC was reported as partially alopecic and covered by crusts [15–18]. Histologically, the neoplastic cells are limited to the epidermis leaving the basement membrane still intact [16]. Surgical excision seems to be curative and no cases of metastasis have been reported so far. However, there are some reports of BISC that were left untreated and progressed to infiltrative SCC [16, 17].

The knowledge about the epidemiology of FdPV2 infections is still limited. Since its discovery, FdPV2 DNA has been found in BISC by various research groups with prevalence ranging from 18 % to 100 %. It was repeatedly amplified from viral plaques. Viral plaques are uncommon, non-neoplastic skin lesions that are clinically indistinguishable from BISC. Although complete regression has been reported, viral plaques are assumed to be precursor lesions of BISC [19, 20]. These studies overall support a causative role of FdPV2 in the development of viral plaques and BISC. However, most of these studies only include small numbers of cats. Furthermore, FdPV2 could also be found in other types of feline skin lesions. As in BISC, the determined prevalence rates of FdPV2 in these other lesions show a rather wide variety, comparable to those found in BISC lesions [4, 5, 18, 19, 21–23]. Only a few studies included samples from cats’ normal skin. Amplification of PV DNA using broad range primers always failed. However, in one study, a set of specific primers was used. FdPV2 DNA could be amplified from 52 % of the samples [24] Furthermore, FdPV2 DNA prevalence in eleven queens and their kittens was reported to be 100 % and 91 %, respectively [25]. This study demonstrated the high prevalence of FdPV2 in the cat population.

There are no reports about the seroprevalence of FdPV2. Indeed, no assay for the measure of FdPV2 specific antibodies has been developed so far. We therefore established a Glutathione-S-Transferase (GST) capture ELISA [26] for detection of antibodies directed against the major capsid protein L1 of FdPV2. We have previously used this technique to determine the seroprevalence of Canine Papillomavirus (CPV) 1 and CPV3 [27] and Equine Papillomavirus 2 (EcPV2) [28] in corresponding populations.

The aim of the present study was to determine the prevalence of FdPV2 in cats that do not suffer from any dermatological conditions. First, the aim was to determine the genoprevalence on skin. Secondly, we wanted to determine the seroprevalence of FdPV2. Finally, the genoprevalence and the seroprevalence of individual cats were compared.

## Methods

### Sampling of cats

With the owners consents, we sampled 125 cats, that were presented to the Clinic of Small Animals, Vetsuisse-Faculty, Zurich, Switzerland. In order to screen “healthy” cats with respect to PV infections. Furthermore, we included only cats without skin diseases nor any conditions impairing the immune system such as hypersensitivity, auto-immunity, neoplasia, immunomodulatory treatments, FeLV, FIV and FIPV infections. From these 100 healthy cats, 60 were male (44 castrated) and 40 were female (20 spayed). Cat ranged in age from three months to 17 years with a median of seven years. Twenty-two cats were less than 1 year old. The age of 10 cats was unknown. Seventy-six cats were mixed breeds and 24 cats were purebred cats or descendants of two different purebred cats, respectively.

Skin cell samples were taken with a Cytobrush cell sampler (Deltalab; Barcelona, Spain). Two samples were taken from each cat. The first sample was taken from the haired skin around the mouth in the area where the left vibrissae are located. The second sample was taken from the right front paw, interdigitally between P3 and P4. If the described areas were not accessible for any reason (e.g. injury or bandage), the corresponding areas on the contralateral side were used for sampling. Briefly, a Cytobrush was wetted in 0.9 % sterile NaCl solution and rubbed with rotating movement for 30 s on the skin of the described area. The handle of the Cytobrush was then cut off and the brush part placed in a sterile 1.5 ml Eppendorf tube.

Serum samples were taken during routine diagnostics not related to our study or when a new intravenous catheter was placed. Animals with a known or suspected history of immunodeficiency or under treatment with immunosuppressive drugs were not included. If a complete blood count of a candidate was available, it was checked and cats suspected immunodeficiency were excluded.

Two cats with lesions that had been histologically confirmed as BISC served as positive controls. The Cytobrush samples were taken directly from the BISC lesions. One cat was sampled at two lesions on the neck whereas the other cat was sampled at one lesion on the forehead so that in a total of three samples were obtained. Serum samples were taken during routine diagnostics.

As a negative control, Cytobrush and serum samples were taken from 5 specific pathogen-free (SPF) cats [29]. The Cytobrush samples were taken from the same locations as described above. All serum and Cytobrush samples were stored at −20 °C until further analysis.

### PCR

DNA was extracted from the Cytobrush samples using QIAamp® DNA Mini Kit (Qiagen; Basel, Switzerland) according to the manufacturer’s protocol but with double amount of buffer ATL, proteinase K, buffer AL and ethanol. The extracted DNA was finally dissolved in 100 μl of buffer AE.

Quantitative real-time PCR (qPCR) was performed using the iCycler iQ™ Real-Time PCR Detection System (Bio-Rad; Hercules CA, USA). Reactions contained 10 μl iQ™ SYBR® Green Supermix (Bio-Rad; Hercules CA, USA), 0.6 μl forward primer (10 μM; fdpv2_qpcr_for: 5′-CAG CTC CCA GTC TCC TAA CG-3′), 0.6 μl reverse primer (10 μM; fdpv2_qpcr_rev: 5′-GCT GTG CCA TTA TCT GAG CA-3′), 3.8 μl sterile water and 5 μl template DNA. Negative controls contained no template DNA but additional 5 μl of sterile water. The following amplification conditions were used: 3 min at 95 °C, 41 cycles of 10 s at 95 °C and 30 s at 60 °C and 1 cycle of 1 min at 95 °C and 1 min at 55 °C. Afterwards temperature was raised by 0.5 °C per cycle during 84 cycles of 10 s to create the melt curve.

As a reference gene, feline glyceraldehyde-3-phosphate dehydrogenase (GAPDH) was chosen. A set of primers (gapdh_qpcr_for: 5′-GTG GAG GGA CTC ATG ACC AC-3′ and gapdh_qpcr_rev: 5′-GTG AGC TTC CCA TTC AGC TC-3′) was designed to amplify cat’s GAPDH. qPCR was performed using the same protocol as described above.

Calibration curves were created with dilution series of plasmid DNA. For FdPV2, the plasmid containing the entire FdPV2 DNA was used. For GAPDH, an amplimer of a PCR reaction (with the primers gapdh_for: 5′-TCA TCA TCT CTG CCC CTT CT-3′ and gapdh_rev: 5′-GTG AGC TTC CCA TTC AGC TC-3′) was cloned, sequenced and then used as template DNA for calibration curve creation.

### Antigen production for ELISA

The FdPV2 L1 coding sequence (CDS), lacking the first ten (5′) codons, was amplified by PCR from the cloned whole genome of FdPV2 [11] using Phusion™ High-Fidelity DNA Polymerase (Finnzymes; Espoo, Finland). Flanking *Bam*HI sites at the ends of the amplimer, introduced by the primers (fdpv2_L1_for: 5′-CGA CGG ATC CTT ATA TCT CCC ACC CTC CCC TG-3 and fdpv2_L1_rev: 5′-AAT AGG ATC CTC ATT TGC GGG TGC GTT-3), facilitated the cloning into the *Bam*HI site of the pGEX-6P-1 vector (Pharmacia Biotech; Uppsala, Sweden). Protein expression in *E.coli* strain BL21(DE3), which express the T7 polymerase upon IPTG induction, was performed as described previously with minor modifications [30]. In brief, bacteria were grown in LB medium containing 100 μg/ml Ampicillin at 25 °C with shaking up to an OD_600_ of 0.3 when protein expression was induced by adding 0.25 mM isopropyl-β-D-thio-galactoside (IPTG) and incubated over night at 25 °C with shaking. Pelleted bacteria were resuspended in 1/10 of the culture volume of buffer L (40 mM Tris pH 8.0, 200 mM NaCl, 1 mM EDTA and 2 mM DTT) supplemented with Complete Protease Inhibitor Cocktail (Roche; Mannheim, Germany) and lysed by sonication. ATP (2 mM) and MgCl_2_ (5 mM) were added and the lysate was incubated for 1 h at room temperature. Urea was slowly added over 5 min to a final concentration of 3.5 M. After incubation of 2 h at room temperature, the mixture was dialysed over night at 4 °C against buffer L using 7 K MWCO Slide-A-Lyzer® Dialysis Cassettes (Thermo Scientific; Rockford IL, USA). After centrifugation the obtained antigen mix was diluted 1:1 with glycerol and stored at −20 °C.

The protein expression procedure was simultaneously performed with three different *E.coli* strain BL21(DE3) cultures containing different pGEX-6P-1 vector derivatives. The first contained the FdPV2 L1 CDS fused to the GST CDS whereas the second contained a CPV1 L1 CDS fused to the GST CDS [27]. The third culture contained the GST CDS only. All ELISA assays reported in this study were performed with antigen from the same lot of antigen production.

### GST capture ELISA

Throughout the protocol, plates were washed three times with PBS buffer supplemented with 0.3 % Tween 20 (PBS-T) between every incubation step. Polysorb 96-well plastic plates (Nunc; Roskilde, Denmark) were prepared for the ELISA. They were coated at 4 °C over night with 50 mM sodium carbonate buffer pH 9.6 containing 0.2 % glutathione casein (kindly provided by Martin Müller DKFZ, Heidelberg, Germany) and then blocked at 37 °C for 1 h with casein buffer (PBS-T containing 0.2 % casein). The GST tagged antigen, diluted 1:10 in casein buffer, was applied to the plates and incubated at 37 °C for 1 h.

Prior to ELISA, the sample sera had been diluted 1:500 in casein buffer, mixed with an equivalent of lysed untransformed *E.coli* strain BL21 (DE3) and incubated at 4 °C for 30 min to block reactions with contaminating bacterial proteins [26, 27, 30]. The sera, cleared by centrifugation, were applied to the plates and incubated at 37 °C for 1 h. Goat Anti-Feline IgG conjugated to Horseradish Peroxidase (HRP) (Southern Biotech; Birmingham AL, USA) diluted 1:1000 in casein buffer was added as secondary antibody and the plates were incubated again at 37 °C for 1 h. After six final washes with PBS-T, substrate (78 mM CH_3_COOH, 24 mM CH_3_COONa, 50 mM NaH_2_PO_4_, 2 mM ABTS [Roche; Rotkreuz, Switzerland] with 1.25 mM H_2_O_2_ applied shortly before use) was added. Absorbance was measured after 45 min at 405 nm in a Sunrise™ microplate reader (Tecan; Männedorf, Switzerland).

The cat sera were tested in triplicates against the antigen FdPV2 L1-GST and, as a negative control, against CPV1 L1-GST. For a subset of samples the ELISA was repeated. The according samples were then tested in duplicates against CPV1 L1 and against GST alone. In order to normalize the results of the different plates, the same positive and negative control sera were used on every plate. No serum was added in six wells serving as a plate control.

### Data analysis and presentation

The C_q_-values obtained from qPCR were converted into absolute numbers of copies of FdPV2 and GAPDH in each sample using the equation of the corresponding calibration curve. The C_q_ values of samples revealing no amplification within forty qPCR-cycles were set to 40 for further calculations. In order to obtain comparable results, in each sample the absolute number of FdPV2 copies was divided by the corresponding absolute number of GAPDH copies.

Serum samples were tested in triplicates in ELISA. To prevent outlier results from influencing the data, the median of the three observed values was used for further analysis. Plate to plate variability was compensated by dividing every value by the mean of the control sera values from the corresponding plate and multiplying the result by the mean of all control sera from all plates.

A cut-off value (COV) was set by the mean of all negative control samples plus two standard deviations. Figures were generated using R (Free Software Foundation; Boston, USA).

## Results

### Genoprevalence of FdPV2

In order to test the skin samples for the presence of FdPV2 specific DNA, a qPCR assay was developed. First, calibration curves for qPCR of FdPV2 and GAPDH DNA were determined (Additional file 1: Figure S1). Second, the cut-off-value (COV) for the ratio of FdPV2 to GAPDH was set as the mean of the negative control samples plus two standard deviations of the mean from the negative control samples. Therefore, the COV was set at 0.367 and the log_2_(COV) at −1.446, respectively. The newly developed qPCR assay was applied for the measurement of viral DNA in the Cytobrush samples collected from the healthy sample population. Eleven samples were excluded from the analysis because an unspecific by-product was amplified or the amplification of GAPDH failed and consequently the calculation of the copy number of FdPV2 per GAPDH was not possible. Out of the 200 DNA samples, 189 could therefore be used for the study representing all cats with at least one sample.

**Fig. 1:**
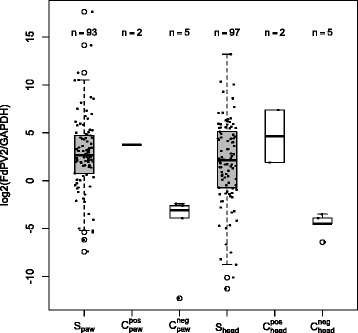
Boxplot of the log-transformed ratios of FdPV2 to GAPDH molecule numbers. The positive controls (C^pos^_paw_, C^pos^_head_) and the negative controls (C^neg^_paw_, C^neg^_head_) are shown as white boxes and the samples (S_paw_, S_head_) as grey boxes. The solid bar represents the median, the box range from the first to the third quartiles, and the whiskers extend to the lowest and highest datum still within 1.5 times the interquartile range. Data not included within the whiskers are individually presented

The genoprevalence within the sample population was evaluated considering the ratios of FdPV2 to GAPDH DNA copies as determined by the analysis of the qPCR-results. The log-transformed ratios are shown as box plots in Fig. 1. The medians of the negative controls were significantly lower than those from the positive controls The values of the negative and positive controls range from 2.0 · 10^−4^ to 8.8 · 10^−2^ and 3.7 · 10^0^ to 1.7 · 10^2^, respectively. The medians of the samples from the head as well from the paw were between the positive and the negative controls. Among the 189 samples used for further analysis, the calculated FdPV2 DNA copies per GAPDH varied from 4.0 · 10^−4^ to 9.3 · 10^4^ in the samples from the head and from 5.9 · 10^−3^ to 2.0 · 10^5^ in the samples from the paw.

No relationship between the copy numbers in head and paw samples could be found. A high number of copies in the head sample was not necessarily accompanied by a high number of copies in the corresponding paw sample and vice versa.

Of the 189 samples used in the analysis, 169 samples were above the COV whereas 20 samples remained below. Of these 20 samples, 13 were taken from the head and seven from the paw. Ninety-eight cats had at least one positive sample and were therefore counted as FdPV2 DNA positive. The two FdPV2 DNA negative cats both had two samples of sufficient quality (according to the exclusion criteria mentioned above) and did not have any relation to each other. Summarized, the DNA prevalence of FdPV2 in the studied population was determined to be 98 %.

### Seroprevalence of FdPV2

In order to test the serum samples for the presence of FdPV2 specific antibodies, an ELISA was developed and applied. First, the antigen coating of the plates and the measurement of antibodies in the control sera were tested. Second, the antibody titres of all samples were determined and normalized. Third, the COV was set as the mean plus two standard deviations of the mean from the negative control samples. Fourth, the seroprevalence of the sample population was determined.

The serum samples were screened for antibodies against FdPV2 and, as a negative control, against CPV1 using a GST capture ELISA. The sample sera reacted against FdPV2 producing an OD ranging from 0.154 to 1.094 (mean = 0.301) and against CPV1 with an OD ranging from 0.162 to 1.096 (mean = 0.242). The COV was set at of 0.315 for FdPV2 and 0.472 for CPV1. The reactions against FdPV2 of 24 serum samples were above the COV and could thus be considered positive while 76 serum samples were counted as negative. Two serum samples showed a reaction against CPV1 above the according COV.

**Fig. 2:**
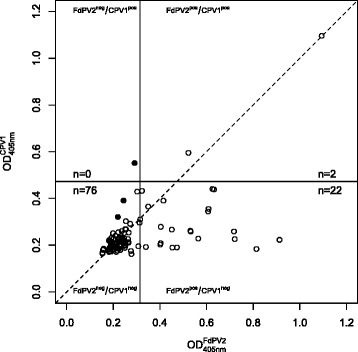
OD values of the FdPV2 specific ELISA versus OD values of the CPV1 specific ELISA. The respective COVs is shown as vertical line for the FdPV2 ELISA and as horizontal line for the CPV1 ELISA. To visualize data points with values of the CPV1 OD above the FdPV2 OD, a diagonal line is drawn. Samples are shown as circles and negative controls as black dots. The positive control is outside the range of the axis

To incorporate the CPV1 control in the data analysis, the corrected OD values of the FdPV2 specific ELISA were plotted against the corrected OD values of the CPV1 specific ELISA (Fig. 2). As mentioned above, two of the 24 positive serum samples showed a reaction against CPV1 with an OD above the according COV (circles on the right side of the vertical and above or close to the diagonal line on Fig. 2). The ELISA was repeated with these samples. CPV1 L1 (tagged to GST) and GST alone were used as antigens. In both samples the reactions against GST alone were as strong as the reactions against CPV1 L1-GST. The samples were therefore categorised as negative for antibodies against CPV1 as well as against FdPV2. The seroprevalence of FdPV2 was corrected down to 22 %.

The OD of the negative control sera ranged from 0.184 to 0.291 (mean = 0.225) and the one of the positive control sera from 1.743 to 2.041 (mean = 1.892) in the FdPV2 specific ELISA. Bonferroni statistical test was used to compare the mean OD of the sample sera with those of the positive and negative control sera, respectively. The mean OD of the positive control sera was significantly higher than the mean OD of the sample sera (*p* > 0.001), whereas the mean OD of the negative control sera did not differ significantly from the mean OD of the sample sera (*p* = 0.986). Fisher’s Exact Test was used to compare subgroups of the sample population. No difference in seropositivity could be found between purebred and mixed breed cats (*p* = 0.386), nor between male and female cats (*p* = 0.448), nor between intact individuals and neutered ones (*p* = 0.061). The seropositive cats had a median age of 12.0 years. This is significantly older (Univariate Analysis of Variance, *p* > 0.001) than the negative cats that had a mean age of 4.3 years. Yet, the age of three positive and seven negative cats was not known.

### Correlation of genoprevalence to seroprevalence

**Fig. 3:**
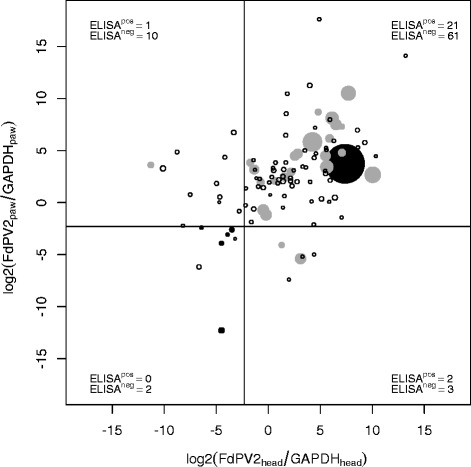
Scatter plot of log-transformed qPCR copy numbers ratios of FdPV2 DNA to GAPDH DNA in head and paw samples and the according ELISA OD value from the serum sample. White circles represent FdPV2 ELISA negative cats from sample population, grey dots represent FdPV2 ELISA positive cats, and black dots represent positive and negative control cats. The drawing sizes of the circles/dots indicate the ELISA OD value. The vertical and horizontal lines are set at the level of the COV (0.202). The numbers of FdPV2 ELISA positive and negative cats in each of the four categories describing the genoprevalence of FdPV2 on head and paw are specified

Results obtained from ELISA and qPCR assay were compared. The log-transformed ratios of the Cytobrush samples isolated from the head were plotted against the ones from the paw and the size of the dot corresponded to the OD value of the FdPV2 specific ELISA (Fig. 3). Data points corresponding to cats with similar ratios of FdPV2 DNA to GAPDH DNA on paw and head would lie close to a diagonal line through the origin with a slope of 1. Such a correlation was not obvious. However there was a tendency of FdPV2 ELISA positive cats within the region of qPCR positive in paw- and head-samples and FdPV2 ELISA negative cats within the region of negative qPCR samples. Some cats had different ratios of FdPV2 and GAPDH DNA in the paw- and head-samples though. Cats tested positive in the ELISA showed a large variation in PCR results. The lowest observed copy number of FdPV2 per GAPDH was as low as 4.0 · 10^−4^ whereas the highest value was 1.4 · 10^3^. Two seropositive cats had less than one copy of FdPV2 per GAPDH in both samples and two other had one negative PCR sample each. None of the positive control samples had more than 200 copies of FdPV2 per GAPDH. The lowest FdPV2 DNA per GAPDH content in a sample from the seronegative cats was 9.0 · 10^−4^, while the highest was 2.0 · 10^5^. One seronegative cat had more than 9.0 · 10^3^ copies of FdPV2 per GAPDH in both samples. The two cats that remained DNA negative were tested negative in ELISA as well. Overall the seronegative cats had on average almost 15 times as many copies of FdPV2 DNA (mean 1.4 · 10^3^) than seropositive cats (mean 1.0 · 10^2^).

## Discussion

Several studies, which evaluate the genoprevalence of PVs in cats are published and listed in Table 1. Most of these papers address the presence of PV DNA in various lesions and only two were done on healthy skin samples. Consistently, the prevalence of DNA in SCC is high while it is hardly reported in unrelated lesions and healthy tissue. Instead of generating a Boolean data set for the genoprevalence, our study assessed the relative amount of FdPV2 DNA to genomic DNA. Such a quantification of viral DNA was previously proposed to rule out a possible causative role of FdPV2 in certain types of lesions since FdPV2 DNA was detected in normal feline skin [24]. Indeed, recently a qPCR-approach was used to quantify the FdPV2 DNA from swap samples of eleven queens and their kittens [25]. In our study we could amplify specific FdPV2 DNA above our threshold from 98 % of the healthy skin samples. This DNA prevalence was thus much higher than most previously reported ones. Only one study detected 52 % [24] and another one 91 % to 100 % FdPV2 DNA positive healthy skin samples [25]. The discrepancy between the various studies might have three primary reasons. First, several different primer sets were used to amplify PV specific DNA in the studies. As a consequence, it is difficult to directly compare the reported prevalences. In particular, only using FdPV2 specific primers but not degenerated ones allow the amplification of FdPV2 DNA [25, 31]. Second, DNA extraction in other studies was partly done from formalin fixed tissue samples. Formalin-fixation tends to degrade DNA, which might lead to a reduced sensitivity of detection [32]. Third, samples from healthy skin were typically collected using cotton-swabs. Indeed, Munday et al*.* showed that amplification of PV DNA from cotton-tipped swabs was a more sensitive method than amplification from formalin-fixed tissue [33]. However, we used Cytobrushes for the collection of cells from the surface of healthy skin. Cytobrushes are intended to take cell samples from mucosae. Chalvardjian et al*.* compared the uptake of cells of cotton-tipped swabs and Cytobrushes by performing endocervical sampling in women. The samples taken with a Cytobrush contained on average at least seventeen times more endocervical cells than the samples taken with a cotton-tipped swab in 87 % of the cases [34]. In our study, only 3 out of 200 Cytobrush samples were negative in qPCR for GAPDH and, therefore, 197 samples were apparently of sufficient quality. This demonstrates that Cytobrushes are a very useful and efficient tool for collecting cell samples even from the haired skin.

**Table 1:** Rates of reported PV DNA findings in skin samples from cats using PCR

Study	Primer set	BISC	Viral plaques	SCC	ISCC	OSCC	Other lesions	Normal skin
Kidney, 2001 [44]	NO1/NO2						0/50 (0 %)	
	E5^+^/E5^−^						0/50 (0 %)	
	IFNR-2/IDNT-2						0/50 (0 %)	
Antonsson, 2002 [41]	FAP59/FAP64							0/5 (0 %)
Nespeca, 2006 [10]	PapF/PapR	1/21 (5 %)			0/22 (0 %)		0/11 (0 %)	
	CP4/CP5/PPF1	5/21 (24 %)			4/22 (18 %)		0/11 (0 %)	
Munday, 2007 [15]	FAP59/FAP64	11/18 (61 %)					0/15 (0 %)	0/3 (0 %)
	IFNR-2/IDNT-2	9/11 (82 %)					0/15 (0 %)	0/3 (0 %)
		*5/18 (28 %)*						
Munday, 2008 [18]	^a^	*20/20 (100 %)*			*17/20 (85 %)*		*3/17 (18 %)*	
Munday, 2008 [33]	FAP59/FAP64		2/2 (100 %)					
	MY09/MY11		2/2 (100 %)					
	JMPF/JMPR		*2/2 (100 %)*					
Lange, 2009 [11]	A16/A37	*3/3 (100 %)*						
Munday, 2009 [31]	FAP59/FAP64					1/20 (5 %)	0/20 (0 %)	
	IFNR-2/IDNT-2					0/20 (0 %)	0/20 (0 %)	
	MY09/MY11					0/20 (0 %)	0/20 (0 %)	
Munday, 2009 [21]	FAP59/FAP64			1/1 (100 %)				
	JMPF/JMPR			*1/1 (100 %)*				
Anis, 2010 [4]	^b^	3/3 (100 %)			5/5 (100 %)	1/1 (100 %)	1/1 (100 %)	
		*3/3 (100 %)*			*4/5 (80 %)*			
Munday, 2009 [24]	MY09/MY11							0/44 (0 %)
	JMPF/JMPR							*34/88 (39 %)* ^c^
Munday, 2010 [19]	MY09/MY11		4/14 (29 %)				0/14 (0 %)	
	FAP59/FAP64		4/14 (29 %)				0/14 (0 %)	
	JMPF/JMPR		*14/14 (100 %)*				*1/14 (7 %)*	
Munday, 2010 [45]	MY09/MY11						4/7 (57 %)	0/120 (0 %)
	jmpSA-F/jmpSA-R						6/7 (86 %)	0/120 (0 %)
Munday, 2011 [22]	MY09/MY11			7/70 (10 %)				
	JMPF/JMPR			*33/70 (48 %)*				
Munday, 2011 [23]	^a^	*14/14 (100 %)*	*8/8 (100 %)*		*12/18 (67 %)*		*1/14 (7 %)*	
Munday, 2011 [46]	FAP59/FAP64					0/30 (0 %)		
	MY09/MY11					0/30 (0 %)		
O’Neill, 2011 [5]	^d^	7/22 (32 %)			11/74 (15 %)		2/12 (17 %)	
		*4/22 (18 %)*			*4/74 (5 %)*			
Schwittick, 2011 [47]	^a^	*1/1 (100 %)*						
Munday, 2013 [12]	FAP59/FAP64	0/1 (0 %)						
	JMPF/JMPR	0/1 (0 %)						
	MY09/MY11	1/1 (100 %)						
	JMY2F/JMY2R	1/1 (100 %)						
Dunowska, 2014 [13]	FAP59/FAP64						0/1 (0 %)	
	MY09/MY11						1/1 (100 %)	
	Unnamed set						1/1 (100 %)	
Thomson, 2015 [25]	E7SF/E7SR							*11/11 (100 %) 20/22 (91 %)*

FdPV2 DNA detection are written in italic

*BISC* Bowenoid in situ carcinoma, *SCC* squamous cell carcinoma, *ISCC* infiltrative squamous cell carcinoma (when defined in study), other lesions = actinic keratosis, allergic dermatitis, apocrine gland cyst, apocrine gland cystadenoma, dermatophytosis, dysplasia, eosinophilic granuloma, eosinophilic plaques, feline leprosy, fibrosarcoma, glossitis, granulomatous dermatitis, hyperplastic tonsil, hypersensitivity dermatitis, mast cell tumour, melanoma, periodontal disease, plasmacytic stomatitis, sarcoids, trichoblastoma, ulcerative gingivitis

^a^nested PCR with FAP59/FAP64 and JMPF/JMPR

^b^HPV1, HPV2, HPV4, HPV7, HPV8, HPV10, HPV15

^c^equals 23/44 cats (52 %)

^d^FAP59/FAP64 and nested PCR with CP65/CP70 and CP66/CP69

In a further set of experiments, we determined the seroprevalence of FdPV2. We found 22 % of the animals positive for FdPV2 specific antibodies in the investigated population of healthy cats. Similar ratios of seropositive animals were detected among healthy horses for antibodies against EcPV2 (28 %) [28], and healthy dogs with antibodies against CPV1 (22 %) or CPV3 (27 %) [27] and koalas (20 %) [35]. Likewise, antibodies against different types of HPV in an Australian human cohort was found - although showing differences depending on age and gender - in the range of 0 % to 22 % positive individuals [36]. Neutralizing antibodies against EcPV2 were detected in 15 % of apparently healthy horses [37]. As negative controls, we used CPV1 L1 GST fusion protein and GST alone. Two of the cat sera reacted as strong to both of these proteins as to FdPV2 GST. GST is an enzyme that plays a key role in cellular detoxification. It is not only present in mammals but also in fungi, helminths and bacteria [38, 39]. Therefore it can be hypothesized that the mentioned two serum samples contained antibodies against GST. Consequently, these sera were not considered positive, neither for FdPV2 nor for CPV1.

Comparing the individual cat’s results from the ELISA and the qPCR assay, a certain correlation might be expected. In humans, individuals with a high HPV DNA load are more prone to be also seropositive [40]. Likewise, contact with FdPV2 might as well induce the production of antibodies against it. Therefore, cats with a high virus load were expected to be more often seropositive. In our study, only a very weak correlation between virus load and seropositivity could be found. Indeed, significantly higher amounts of FdPV2 DNA were detected in some seronegative cats.

Asymptomatic PV infections are quite common. Up to 80 % of humans are infected asymptomatically with human PVs [18] and various animal species have also been investigated regarding such PV infections [25, 41–43]. Though, the prevalence varies from species to species. Interestingly, primates showed prevalence rates similar to that of humans [41]. Prior to the amplification of FdPV2 from the normal skin of cats, asymptomatic infections were not considered to be common. The “hit-and-run” carcinogenesis model was used to explain transformation of normal skin cells into neoplastic cells by transient PV infection [15]. It is not clear if the extracted PV DNA originates from infected epithelium cells or from virions that were attached to the skin’s surface. Assuming the amount of asymptomatically infected animals based only on PCR results might therefore be inaccurate. However, it can be stated that 98 % of the cats had certainly been in contact with FdPV2. Considering the high tenacity of PV particles, it might be hypothesized that high amounts of virus are shed to build up an infectious virus reservoir on biological surfaces.

In our study the seropositive cats were significantly older than the seronegative cats. This correlates well with the existing data about BISC being more common in old cats [16, 17]. Seroconversion and disease development does not seem to be dependent on the virus load alone. This weakens again the hypothesis of a straightforward role of FdPV2 causing BISC. The virus might as well be a part of the normal skin flora of cats. The immune system would then not produce antibodies against it as long as the skin is intact. Suggesting that older cats have had more skin traumas in their lives, might further support this theory.

So far, the causative role of FdPV2 in the development of BISC has only been supported by repeatedly amplifying FdPV2 DNA from BISC samples. Here, we add one more argument for this, which may also be used as a clinical marker, namely the observation of an elevated antibody response going along with the development of malignancies. The very high genoprevalence found in our and in the Thompson study [25] implies that the virus is widespread in the cat population, irrespective of the health status of the cat. Thus, it is still possible that FdPV2 represents just a simple bystander of another yet unrecognized causative agent. Furthermore, we are not able to distinguish between intracellular DNA and viral nucleic acid just laying naked or within virions on the surface of the skin. Collection of different samples representing specifically the skin surface or skin tissue from the same patient is desirable. As such, multiple tests can be done and the results can be compared to reveal the replicative state of the viral genomes detected by PCR. Moreover, patients should be sampled repeatedly at different points in time to gain information about the interaction of the virus with the cat’s body. Studies with large sample population are though needed in order to receive reliable data. Finally, this would allow to obtain a better comprehension of the virus’ epidemiology.

## Conclusion

The observed genoprevalence of FdPV2 in healthy cats was 98 %, while the seroprevalence was 22 %. Cats that carry the virus on their skin mount only rarely an antibody response. It might be hypothesized that though the virus is highly prevalent in the cat’s population, it rarely leads to an immune response due to the fact that it is not productively replicating or the replication is sheltered from the exposure to the immune system.

## Additional file

Additional file 1: Figure S1. Calibration curves for qPCR. Dilution series of cloned feline GAPDH DNA amplimer and FdPV2 DNA were used as template and qPCR was performed using specific primer sets. The resulting equations of the calibration curves are shown. These equations were used to quantify the results of the qPCR using the same primer sets but the DNA extracted from the Cytobrush samples as templates. (PDF 572 kb)
