# Supplementary figures and images for: Geno- and seroprevalence of Felis domesticus Papillomavirus type 2 (FdPV2) in dermatologically healthy cats
Source: BMC Vet Res. 2016 Jul 22;12:147. doi: 10.1186/s12917-016-0776-7 (PMC4957317; doi:10.1186/s12917-016-0776-7)

## GAPDH

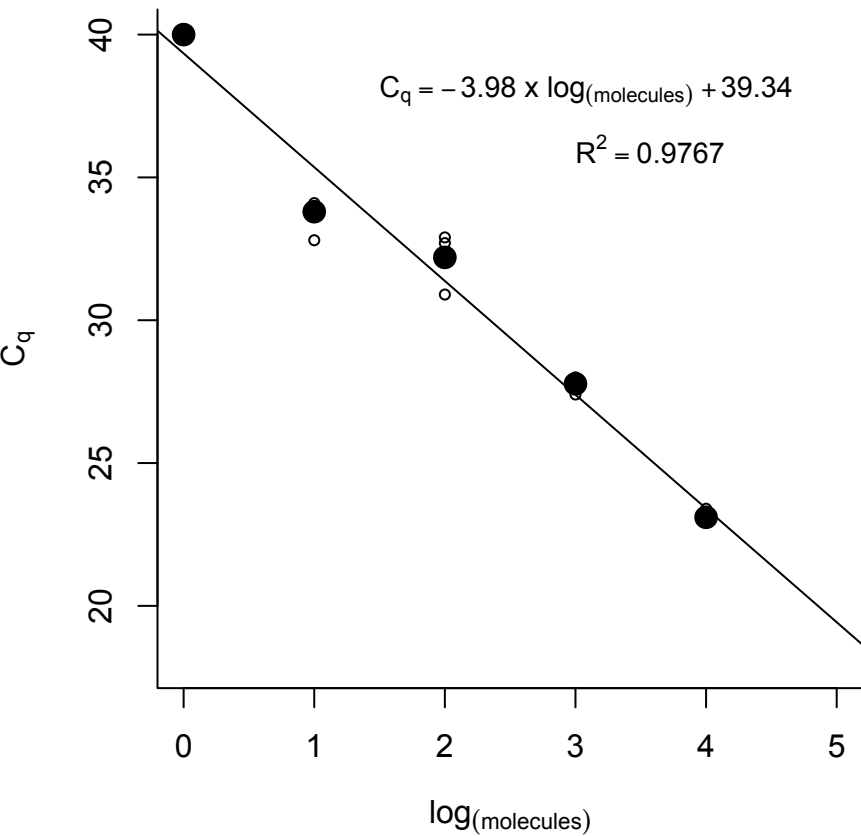

## FdPV2

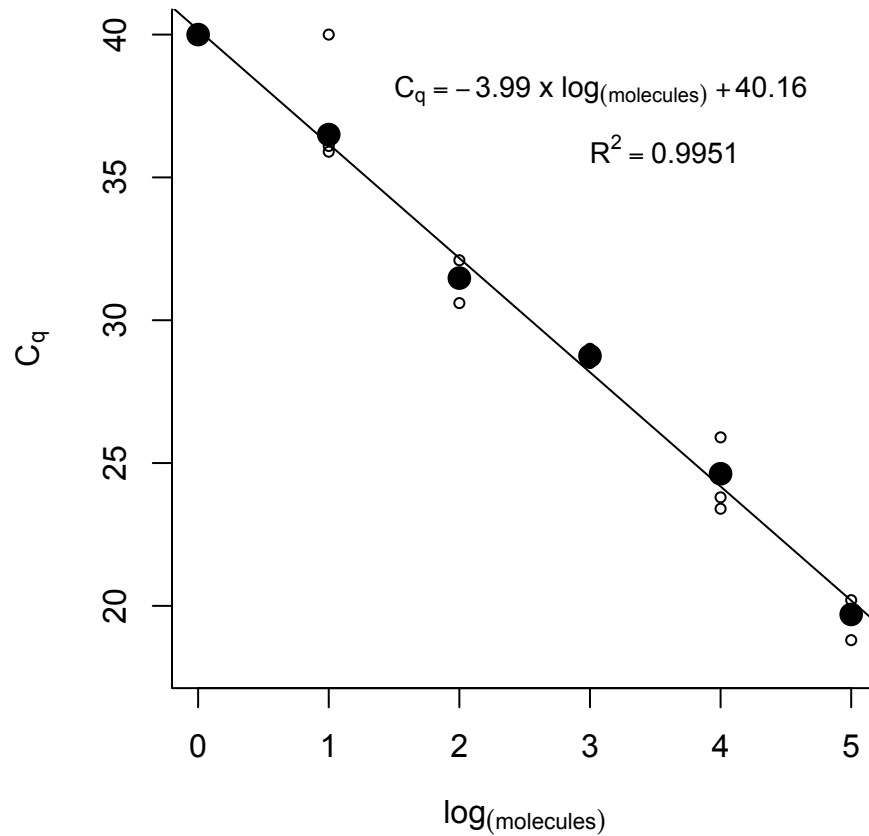

Supplement: Additional file 1: Figure S1. — Calibration curves for qPCR. Dilution series of cloned feline GAPDH DNA amplimer and FdPV2 DNA were used as template and qPCR was performed using specific primer sets. The resulting equations of the calibration curves are shown. These equations were used to quantify the results of the qPCR using the same primer sets but the DNA extracted from the Cytobrush samples as templates. (PDF 572 kb) [file 12917_2016_776_MOESM1_ESM.pdf]
